# Nonreciprocal Electro‐Optic Intermodal Scattering With Momentum Engineered RF Waves

**DOI:** 10.1002/nap2.70170

**Published:** 2026-06-19

**Authors:** Jieun Yim, Gwan In Kim, Violet Workman, Seho Kim, Omar A. Barrera, Ruochen Lu, Gaurav Bahl

**Affiliations:** ^1^ Department of Mechanical Science & Engineering University of Illinois at Urbana–Champaign Urbana Illinois USA; ^2^ Department of Electrical & Computer Engineering University of Illinois at Urbana–Champaign Urbana Illinois USA; ^3^ Department of Physics University of Illinois at Urbana–Champaign Urbana Illinois USA; ^4^ Department of Electrical & Computer Engineering The University of Texas at Austin Austin Texas USA

## Abstract

Spatiotemporal modulation approaches have been often employed as alternatives for producing optical nonreciprocity without magneto‐optic materials. Unidirectional inter‐modal scattering, enabled by either acousto‐optic or electro‐optic (EO) modulation, is a promising method in this category as it can directly modify optical dispersions and even enables linear nonreciprocal photonic devices in the strong coupling limit. Although EO approaches are often preferred for their practicality, it is challenging to generate the large spatiotemporal momentum required for inter‐modal phase matching without EO drive schemes involving multiple drive stimuli. Here, we demonstrate highly selective nonreciprocal inter‐modal EO scattering enabled by a single high‐index radiofrequency (RF) traveling wave stimulus. Our experimental demonstration is performed on a thin‐film lithium niobate‐integrated photonics platform in which we engineer a slow‐wave radiofrequency (SWRF) transmission line with an effective RF index >9 that natively generates the required RF momentum while simultaneously maintaining strong RF‐optical mode overlap. By additionally engineering the interaction length, we achieve a directional ∼20 dB nonreciprocal scattering contrast. The SWRF architecture provides a scalable route to magnetic‐free nonreciprocity and establishes momentum‐engineered RF waves as a powerful tool for next‐generation, fully integrated nonreciprocal photonic systems.

## Graphical abstract

We demonstrate highly selective nonreciprocal intermodal scattering in thin‐film lithium niobate using slow‐wave RF transmission lines. This electro‐optic momentum engineering approach enables magnet‐free nonreciprocal devices with a single RF input.

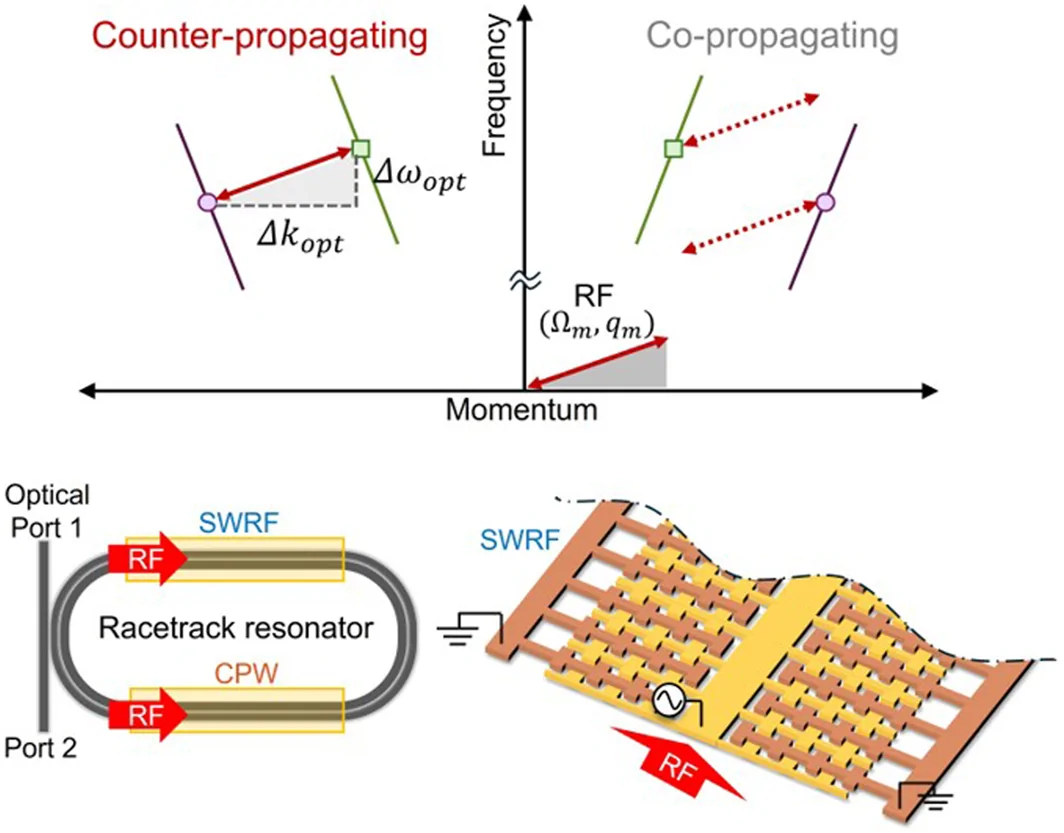

## 1 Introduction

Nonreciprocal components such as isolators and circulators are essential building blocks in photonic systems. However, their on‐chip integration is challenging due to the unavailability of Faraday rotator (i.e., magneto‐optic) materials [1, 2] in photonics foundries. Although there have been some research successes in incorporating magneto‐optic materials into photonic integrated circuits [3, 4, 5], more broadly accessible and foundry‐compatible approaches are still needed—such as spatiotemporal modulation [6, 7, 8, 9, 10, 11, 12, 13]. In this context, unidirectional inter‐modal coupling has emerged as a promising alternative approach for breaking optical reciprocity [14, 15, 16, 17]. In this mechanism, two optical modes on distinct dispersion branches have frequencies and momenta $\left(\omega_{1},k_{1}\right)$ and $\left(\omega_{2},k_{2}\right)$, which are separated by ($\Delta \omega_{\text{opt}}$, $\Delta k_{\text{opt}}$) (Figure 1a). A traveling‐wave stimulus can couple the modes when its frequency $\left(\Omega_{m}\right)$ and momentum $\left(q_{m}\right)$ match $\Delta \omega_{\text{opt}}$ and $\Delta k_{\text{opt}}$, respectively, thereby satisfying the phase‐matching condition. Since $\Delta k_{\text{opt}}$ reverses its sign for the opposite optical propagation direction, a fixed stimulus satisfies the phase‐matching condition and produces inter‐modal coupling in only one optical direction. This approach can be implemented through spatiotemporal modulation in both waveguides and resonators, by means of either the acousto‐optic [18, 19] or electro‐optic effect [12, 17, 20]. Based on this principle, several architectures have been employed to build nonreciprocal devices [6, 7, 8, 12, 18, 19, 20].

**FIGURE 1:**
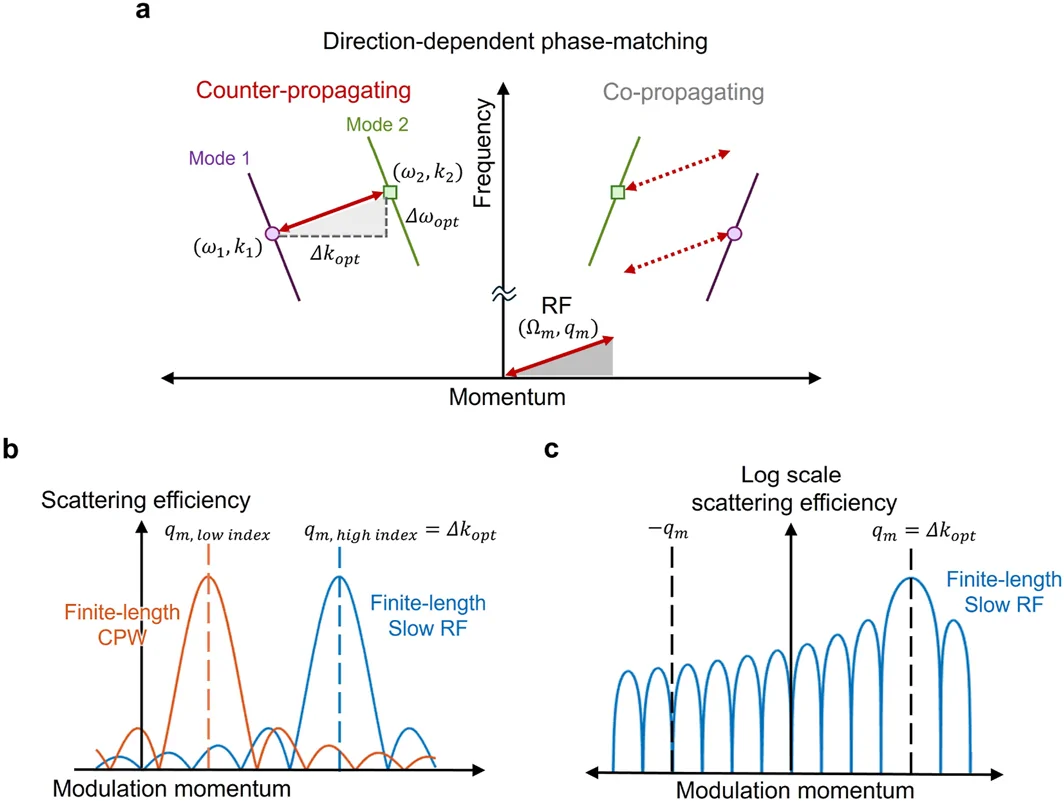
Principle of nonreciprocal inter‐modal scattering by slow RF waves. (a) Diagram for direction‐dependent phase matching of inter‐modal scattering. Two optical modes can be coupled when their frequency difference $\Delta \omega_{\text{opt}}$ and momentum difference $\Delta k_{\text{opt}}$ match the frequency $\Omega_{m}$ and momentum $q_{m}$ of the RF wave, respectively. In the specific configuration shown here, the directionality of the RF wave is only phase‐matched with optical modes in the counter‐propagating direction. In the co‐propagating case, the phase‐matching condition is not satisfied, and the scattering is suppressed. (b) Spectral scattering efficiency at a fixed frequency as a function of modulation momentum for a finite‐length low‐index CPW (orange) and a finite‐length slow RF wave (blue). (c) Same as (b), but plotted on a logarithmic scale, highlighting that the interaction length of slow RF wave can be intentionally designed such that the scattering efficiency exhibits a null at $-q_{m}$, thereby maximizing nonreciprocity.

A key requirement for inter‐modal scattering is a modulation stimulus with the correct momentum to bridge the optical momentum gap $\Delta k_{\text{opt}}$, which tends to be large between distinct mode families. Acoustic waves have low phase velocity and can naturally provide the required momentum at modest frequencies [6, 7, 18, 19, 21, 22]. However, acousto‐optic implementations typically require free surfaces or released structures, which complicates photonic integration, and scaling to higher acoustic frequencies is limited by fabrication constraints on the RF–acoustic transducers. Therefore, direct spatiotemporal modulation via the electro‐optic (EO) effect has come to be of interest, as EO devices behave as capacitive electrical loads, can be fully cladded, and are readily scalable to high RF frequencies.

A major challenge, however, is that the RF phase velocity tends to be high in most coplanar waveguides (CPWs), which leads to a very low momentum at modest GHz frequencies (<100 GHz), making it difficult to satisfy the inter‐modal phase‐matching requirement. One solution to address this issue is to employ a multi‐phase or multi‐drive split‐electrode system [12, 20, 23] to synthetically engineer a large effective momentum that is determined by the electrode pitch. However, it is very challenging to scale this approach to longer modulators or to higher RF stimulus frequencies since small deviations from the required phasing at each electrode can degrade the synthesized RF wave. Such phasing variations can readily arise from manufacturing tolerance issues, leading to capacitance variations, undesirable reflections, standing waves, and other RF engineering concerns. Consequently, scalable electro‐optic nonreciprocal devices may greatly benefit from an EO modulation scheme that is capable of natively generating a propagating wave with large momentum using only a single RF stimulus.

In this work, we address this challenge by exploring a “slow‐wave” radiofrequency (SWRF) transmission line structure [24, 25, 26, 27] whose RF index can be adjusted while, in principle, simultaneously maintaining a desired waveguide impedance. Slow‐wave electrodes have been used in Mach–Zehnder electro‐optic modulators along with slotted and T‐rail electrodes [28, 29, 30, 31, 32, 33, 34] in which the goal is to match the optical group velocity with the RF phase velocity for intra‐modal scattering. However, inter‐modal scattering poses a more challenging problem, as it requires a transmission line with a very high RF index (low phase velocity) that simultaneously provides strong mode overlap between the RF field and two distinct optical modes [18, 35]. Through the approach presented in this work, we address these challenges and demonstrate highly selective nonreciprocal inter‐modal electro‐optic scattering enabled by a single‐drive SWRF transmission line.

## 2 Results

As illustrated in the dispersion diagram (Figure 1a), we consider an optical waveguide system where inter‐modal scattering is phase‐matched when the optical and RF waves are counter‐propagating. We note that this conclusion is design‐dependent and that the phase‐matching may also be satisfied in the co‐propagating direction with a suitable dispersion design. When the interaction length of the optical and RF waves is finite, the strict momentum‐matching condition is relaxed, resulting in a sinc‐shaped momentum response that peaks at $q_{m}$ (see Supporting Information S1: S1). Figure 1b presents an illustrative comparison of this sinc‐shaped scattering efficiency of a finite‐interaction‐length system driven by low‐index (high phase velocity) and high‐index (low phase velocity) RF waves at a fixed frequency $\Omega_{m}$. As the RF stimulus momentum $q_{m}$ and index $n_{\text{RF}}$ are related by $q_{m}=2\pi n_{\text{RF}}\Omega_{m}/c$, we find that a higher index RF transmission line naturally produces a larger momentum for the same stimulus frequency $\Omega_{m}$. Figure 1c explores the scattering efficiency of the high‐index case on a logarithmic scale, where the counter‐propagating and co‐propagating cases, corresponding to $q_{m}=\Delta k_{\text{opt}}$ and $q_{m}=-\Delta k_{\text{opt}}$, respectively, are indicated. Here, we note that it is possible to intentionally engineer the sinc‐shaped spectral response to simultaneously place a maximum at $\Delta k_{\text{opt}}$ and a null at $-\Delta k_{\text{opt}}$ by choosing an appropriate interaction length $L=p\pi /q_{m}$ for any positive integer p, which maximizes the nonreciprocal scattering contrast (see Supporting Information S1: S2). In practice, RF attenuation along the transmission line sets the upper bound on the achievable contrast (see Supporting Information S1: S3).

Our experimental demonstration was conducted in a 600‐nm X‐cut thin film lithium niobate on insulator (LNOI)‐integrated photonics platform at 1550 nm (fabrication details are provided in Methods). We designed a double optical waveguide system (Figure 2a) that supports fundamental TE modes near 1550 nm with odd and even symmetry (Figure 2b) propagating along the crystal $\hat{y}$ axis of the lithium niobate. This dual waveguide approach enables more fabrication‐tolerant control over the momentum gap between the optical modes. The buried oxide was 2 μm thick and we deposited a 2 μm cladding oxide above the optical structures. Gold electrodes in a ground‐signal‐ground transmission line configuration were fabricated above the cladding to produce a mirror symmetric RF electric field for modulation (Figure 2c). With this choice of propagation direction and crystal orientation, the mirror symmetric RF modulation field maximally utilizes the largest Pockels coefficient of lithium niobate ($r_{33}\approx 30$ pm/V) to couple the $\hat{y}$ propagating TE optical modes [12]. The transverse geometry of the electrodes (e.g., gap between the ground and the signal and the thickness of the cladding oxide) was designed to maximize the RF–optical overlap integral while minimizing optical loss induced by the metal electrodes.

**FIGURE 2:**
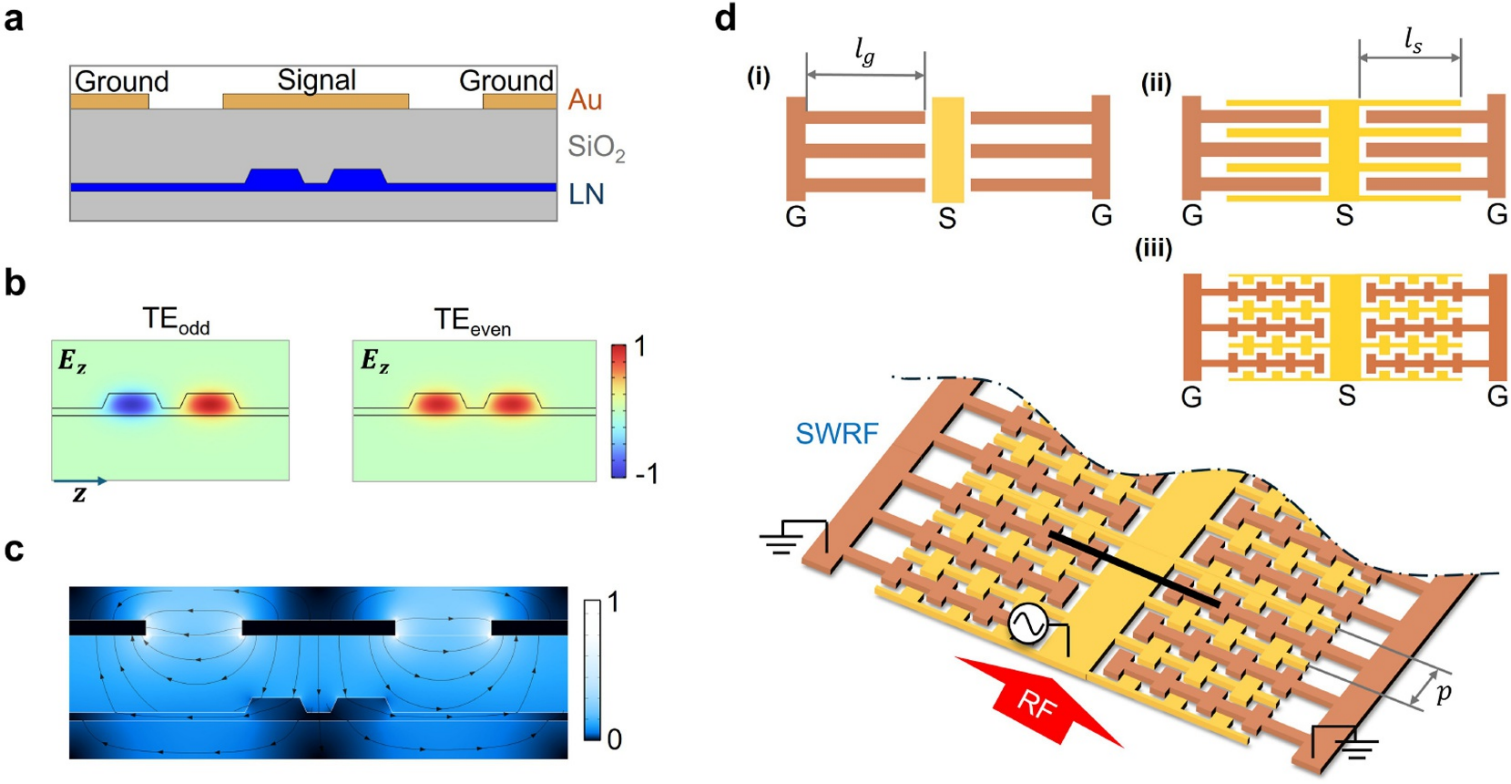
Design and implementation of our experimental device. (a) Cross‐section schematic of the double optical waveguide system and the electro‐optic modulation electrodes. (b) Simulated electric‐field profiles $\left(E_{z}\right)$ of the TE_odd_ and TE_even_ optical modes supported in the waveguide. (c) Simulated radiofrequency field distribution from the electrodes. (d) Schematic of the SWRF electrode. The insets (i)–(iii) illustrate the intermediate design steps of the SWRF electrode (bottom). The structure has a periodicity of p=10 μm. The ground lines (G) have lateral slots of width $l_{g}=170$ μm. The signal lines (S) have lateral extensions of width $l_{s}=36$ μm. The black solid line indicates the location of the cross‐section whose RF field distribution is shown in (c).

The most important component in our experiment is the SWRF transmission line that respects the mirror symmetry with a strong mode overlap. The transmission line inductance L and capacitance C per unit length provide two degrees of freedom to design both the modulation momentum $q_{m}$ and the characteristic impedance $Z_{0}$ according to $q_{m}=2\pi \Omega_{m}\sqrt{LC}$ and $Z_{0}=\sqrt{L/C}$. Our final SWRF transmission line design is illustrated in Figure 2d, with the propagation direction indicated by the red arrow. The structure is composed of a periodic unit cell (with period p = 10μm), which features minimally interrupted straight sections that produce a local RF field (as visualized in Figure 2c) to match a quasi‐TEM mode of a conventional CPW transmission line. This region is indicated with the black cross‐section line in Figure 2d. We manually derived the SWRF structure starting from a standard CPW line by increasing the inductance per unit length L with the introduction of lateral slots of length $l_{g}$ [31] on the ground lines (inset (i) in Figure 2d) and increasing C by introducing lateral extensions on the signal line with length $l_{s}$ (inset (ii) in Figure 2d). At this stage, the resulting GSG transmission line appears as an interdigitated structure. Finally, the signal and ground extensions were patterned with alternating wide and narrow sections, rather than a uniform width, to increase the capacitance without further lengthening $l_{s}$ (inset (iii) in Figure 2d). The final geometry (details in Supporting Information S1: S5) was optimized using full‐wave simulations in Ansys HFSS and COMSOL Multiphysics, yielding an RF effective index of 9.8 and a characteristic impedance of $Z_{0}=42\;\Omega$ over the frequency range $\Omega_{m}=16$–20GHz. This frequency range was chosen based on our experimental constraints on RF drive power, frequency, and measurement bandwidth, but the same design principles are readily applicable at higher RF frequencies. Although the impedance can be further tuned by increasing $l_{g}$, the present design was selected to remain compatible with the footprint of the optical structure. With the estimated RF index in this frequency range, the SWRF electrode should provide a modulation momentum peak near $q_{m,SWRF}=3300$–4100rad/m.

In order to directly compare the scattering efficiency of SWRF and CPW electrodes, we wrapped the dual‐waveguide system into a double‐racetrack resonator that supports the required TE_even_ and TE_odd_ modes, and fabricated both SWRF and CPW electrodes on the identical straight sections (schematic in Figure 3a and image in Figure 3b). A bus optical waveguide interfaces light into the resonator from edge couplers. The free spectral range (FSR) of the resonator was designed to be ∼18 GHz so that we can select and scatter nonadjacent TE_even_ and TE_odd_ modes separated by nearly one FSR (as later shown in the experiment). This enables inter‐modal modulation at $\Omega_{m}$ within the desired 16–20 GHz range. The dispersions of the ${\text{TE}}_{\text{even}}$ and ${\text{TE}}_{\text{odd}}$ modes were then engineered by adjusting waveguide geometry such that the inter‐modal momentum separation $\Delta k_{\text{opt}}$ substantially overlaps with the achievable modulation momentum range ($q_{m,SWRF}$ described above), while remaining compatible with design and fabrication constraints. The final geometry is detailed in Supporting Information S1: Figure S4. For an optical frequency separation range of $\Delta \omega_{\text{opt}}=$ 16–20 GHz, the optimized dual‐waveguide geometry exhibits $\Delta k_{\text{opt}}$ spanning 3190–4190 rad/m between the ${\text{TE}}_{\text{even}}$ and ${\text{TE}}_{\text{odd}}$ mode branches. For reference, the intra‐modal momentum separation between adjacent resonant modes within each mode family is 758–951 rad/m, across the entire laser tuning range of 1520–1570 nm.

**FIGURE 3:**
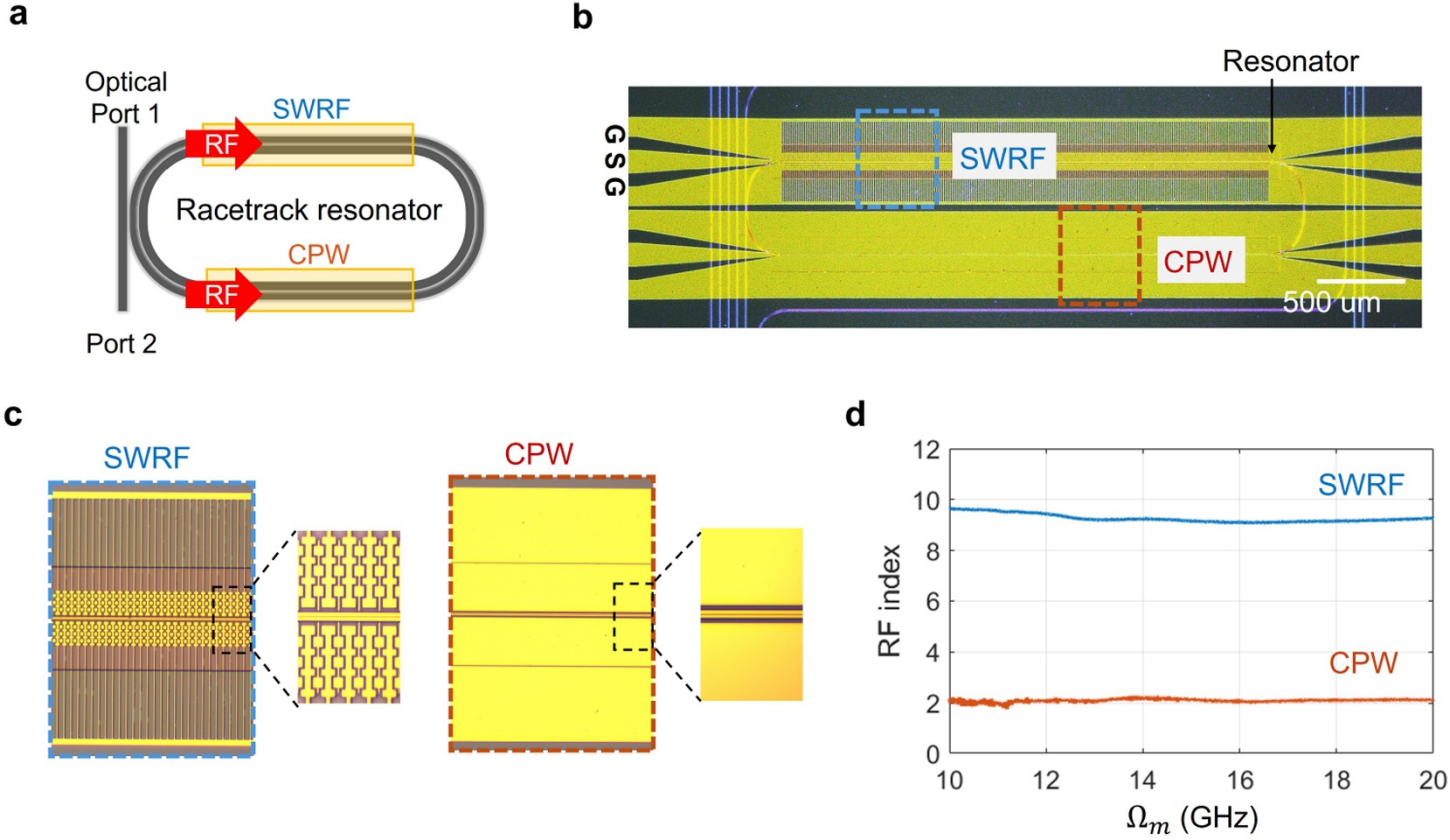
Images of the test device and RF characterization results. (a) Schematic for the device consisting of a racetrack resonator and two modulators (SWRF and CPW electrodes) on two straight sections of the resonator. The optical signal enters through Port 1 and exits through Port 2, whereas the RF signals are launched from the ends of the electrodes. (b) Optical microscope image of the fabricated device. Ground‐Signal‐Ground (GSG) pads are implemented at the ends of the electrodes via adiabatic tapers to interface with the RF input and output signals. (c) Zoomed‐in microscope images of the fabricated electrodes. The left panel shows the SWRF electrode with a periodic interdigitated structure, whereas the right panel shows the CPW. (d) Extracted RF effective index as a function of RF frequency $\Omega_{m}$, showing that the SWRF waveguide achieves a significantly higher index (above 9) compared to the CPW (around 2) over the 10–20 GHz range.

On the straight sections of the racetrack resonator, the SWRF and CPW modulator lengths were set to L=2.85mm. This value was determined (see Supporting Information S1: S2) by considering the simultaneous positioning of the peak and nulls in the momentum spectrum (described previously in Figure 1c) to aim for >20 dB scattering contrast over the targeted range of $q_{m,SWRF}$. We note that, for a fixed value of $q_{m,SWRF}$, multiple interaction lengths L can satisfy the nulling condition. Among these solutions, we selected the longest interaction length that could fit within the straight section of the resonator, which is limited by the FSR constraint.

The RF drive signals were supplied via a standard ground‐signal‐ground (GSG) configuration, with a 50‐Ω load impedance applied on the opposite GSG pads to arrange for a traveling wave excitation. The GSG pads interface with the corresponding transmission lines via an adiabatically tapered section (Figure 3b) to mitigate undesirable reflections. The relative direction of optical and RF signals can be readily switched by reversing the RF input and load or by reversing the direction of input to the bus optical waveguide. We experimentally measured the index of the transmission lines using S‐parameter analysis with a vector network analyzer. We used the general line‐line method (see Supporting Information S1: S6) to extract the propagation constants by comparing the wave–cascade matrices of transmission lines of different lengths over a broad frequency range. The RF index thus measured over the 10–20 GHz range is shown in Figure 3d, confirming that the effective index of our CPW is ∼2, whereas the index of the SWRF electrode reaches ∼9.2 across the measured range, which is slightly lower than our simulated value.

We characterized the nonreciprocal scattering in our device by measuring the generated optical sidebands under RF modulation. We used a heterodyne detection setup (described in Methods) to measure the carrier, Stokes (down‐converted), and anti‐Stokes (up‐converted) signals independently, while sweeping the laser input frequency with detuning over a 26‐GHz span around the modes of interest. Figure 4a shows a carrier transmission spectrum near 1547 nm through the optical bus waveguide and presents the set of optical modes used for subsequent experiments. The ${\text{TE}}_{\text{even}}$ and ${\text{TE}}_{\text{odd}}$ modes have similar intrinsic quality factors of $\approx 6\times 10^{5}$ and are identified by their relative coupling to the bus waveguide. In our double‐racetrack resonator, the ${\text{TE}}_{\text{even}}$ modes couple more strongly to the waveguide, resulting in a greater resonance dip. Figure 4b illustrates the organization of these modes in frequency–momentum space, as inferred from the transmission spectrum and our design of the dispersion.

**FIGURE 4:**
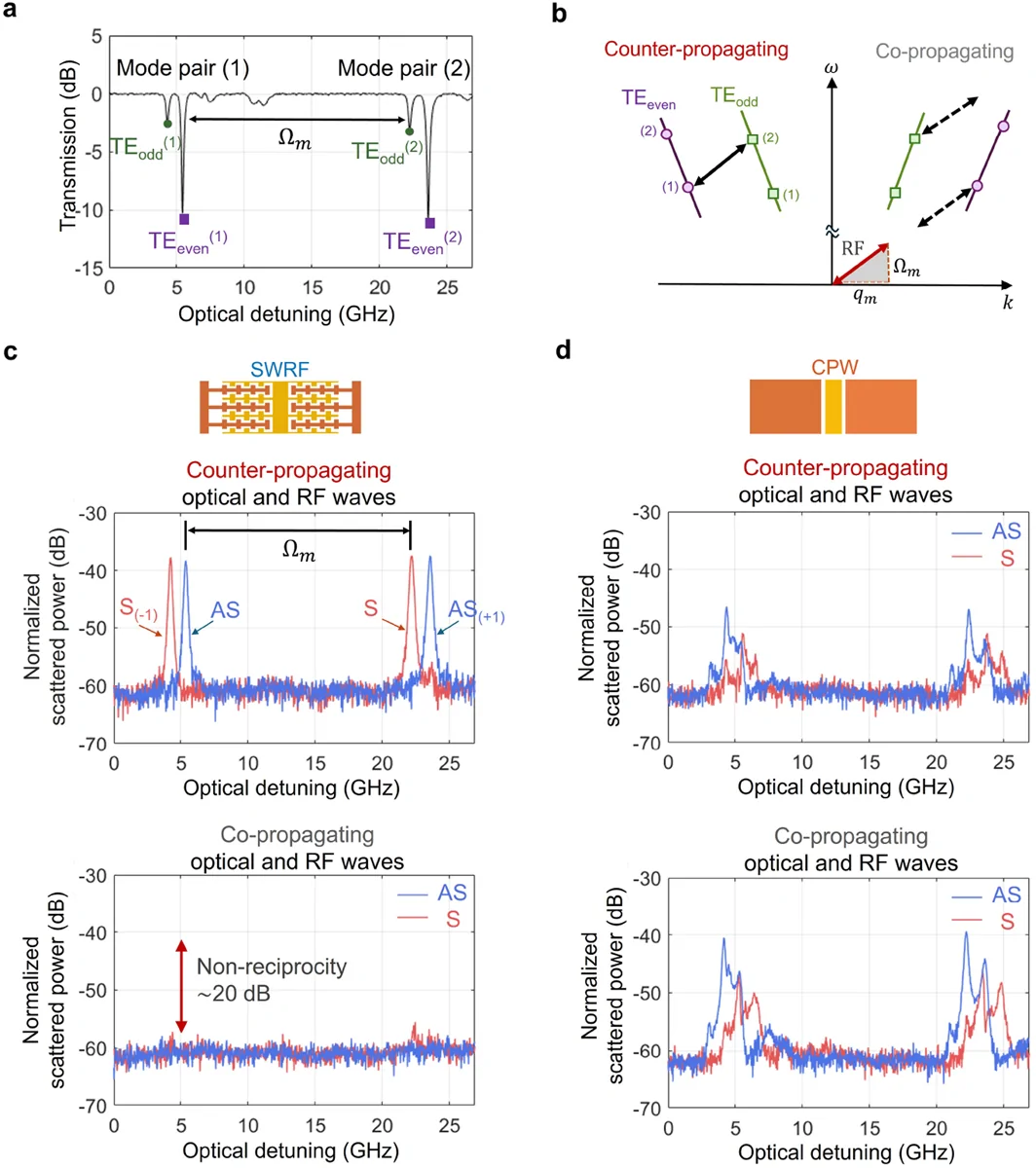
Experimental characterization of nonreciprocal scattering. (a) Measured optical transmission spectrum of the racetrack resonator, showing two mode pairs (Mode pair (1) and Mode pair (2)) separated by one FSR. The inner mode pair TE_even_^(1)^ and TE_odd_^(2)^ are separated by 16.8GHz, corresponding to the RF modulation frequency $\Omega_{m}$ used in this study. (b) The inferred positioning of the optical modes is indicated in this diagram, along with inter‐modal scatterings enabled by the RF stimulus. (c) Measured and normalized scattered optical power under SWRF modulation. When the optical and RF signals are counter‐propagating (top), strong nonreciprocal inter‐modal scattering is observed, with well‐resolved anti‐Stokes (AS) and Stokes (S) sidebands. ${\text{AS}}_{\left(+1\right)}$ is the anti‐Stokes scattering to the next FSR, and $S_{\left(-1\right)}$ is the Stokes scattering to the previous FSR. When the optical and RF signals are co‐propagating (bottom), scattered power is suppressed to the noise floor, demonstrating ∼20dB nonreciprocity. (d) Measured and normalized scattered power using the CPW modulator. In both counter‐ and co‐propagating cases, we observe a cluttered spectrum with indications of mixture of inter‐modal and intra‐modal scattering. The RF power delivered to the transmission line was approximately 14.59 dBm for the SWRF transmission line and 14.2 dBm for the CPW transmission line, as estimated from reflection measurements. Sideband powers were normalized to account for this power difference.

To measure the inter‐modal scattering efficiency, we selected the inner mode pair labeled ${\text{TE}}_{\text{even}}^{\left(1\right)}$ and ${\text{TE}}_{\text{odd}}^{\left(2\right)}$, which has a frequency separation of 16.8GHz. The RF drive frequency was therefore fixed at $\Omega_{m}=16.8\;GHz$. During the modulation, the anti‐Stokes and Stokes sideband powers were measured as a function of the input laser frequency. At this drive frequency, the SWRF transmission line supplies a modulation momentum of 3240 rad/m, which is slightly below the targeted range of $q_{m,SWRF}$. Despite this discrepancy, efficient inter‐modal scattering in the phase‐matched direction is still achievable for the designed optical dispersion, with a theoretically predicted nonreciprocal contrast that remains larger than 18.8dB in the sideband powers across the wavelength range of 1520–1570 nm (see Supporting Information S1: Figure S7a).

Experimental results presented in Figure 4c show scattered sideband powers for the co‐propagating and counter‐propagating cases (indicated by black arrows in Figure 4b) using the SWRF transmission line. When the slow‐wave RF drive is applied in the counter‐propagating direction relative to the optical input (upper plot in Figure 4c), the inter‐modal phase‐matching condition is satisfied, which leads to clearly distinguished anti‐Stokes (AS) and Stokes (S) signal peaks. Specifically, a prominent anti‐Stokes offset signal appears when ${\text{TE}}_{\text{even}}^{\left(1\right)}$ is excited, indicating large coupling to ${\text{TE}}_{\text{odd}}^{\left(2\right)}$ through the electro‐optic modulation. This inter‐modal coupling is further confirmed by the presence of the Stokes offset signal when the laser input excites the ${\text{TE}}_{\text{odd}}^{\left(2\right)}$ resonance. In contrast, when the slow‐wave RF drive is applied in the co‐propagating phase‐mismatched direction relative to the optical signal (lower plot in Figure 4c), the scattered power is below our measurement noise floor, implying a scattering nonreciprocity of at least 20dB between the co‐ and counter‐propagating cases. Based on the analysis in Supporting Information S1: S3, we can estimate that the actual nonreciprocity should be around 34 dB if the phase matching is perfect, limited by the RF propagation loss, but this is not experimentally testable due to the noise floor limitations of our setup. We note the existence of two additional scattering peaks in the phase‐matched (counter‐propagating) case. The Stokes signal at TE_odd_^(1)^ (labeled $S_{-1}$) appears due to scattering into the TE_even_ mode that is one FSR lower in frequency. Similarly, the anti‐Stokes signal at TE_even_^(2)^ (labeled $AS_{+1}$) appears due to scattering into the TE_odd_ mode that is one FSR higher in frequency.

To compare against a lower‐index RF stimulus, we repeated the measurements with the same drive frequency, $\Omega_{m}=16.8\;GHz$, now applied to the CPW transmission line. In both the counter‐propagating (upper plot in Figure 4d) and co‐propagating (lower plot in Figure 4d) configurations, the measured response consists of complicated overlapping spectral features that are not cleanly resolved. In the ideal limit of purely intra‐modal scattering, the Stokes and anti‐Stokes sidebands would completely overlap with a spacing determined by the resonator FSR since the availability of optical modes for intra‐modal scattering is symmetric and leads to symmetric up and down conversion. The partially overlapping spectral features here in Figure 4d therefore indicate the simultaneous presence of intra‐ and inter‐modal processes under CPW modulation. By contrast, the distinctly separated sidebands observed under SWRF drive (Figure 4c) are characteristic of highly selective inter‐modal scattering.

In order to better understand these spectral features, we consider the sinc‐shaped momentum spectrum of our finite length CPW waveguide. We calculate that the momentum spectrum peaks at $q_{\text{m,CPW}}=704\;rad/m$ which is much smaller than the inter‐modal momentum separation $\Delta k_{\text{opt}}$ (see Supporting Information S1: Figure S7b).

Consequently, the two relevant optical momentum spacings $\pm \Delta k_{\text{opt}}$ lie on higher‐order lobes of the sinc spectrum, where the scattering efficiency has lower asymmetry. At the same time, because $q_{\text{m,CPW}}$ is close to the intra‐modal momentum separation, the CPW drive efficiently excites intra‐modal scattering. The coexistence of these two mechanisms therefore leads to various interference effects and a cluttered scattering spectrum. In conclusion, these results demonstrate that a slow‐RF stimulus providing sufficiently large modulation momentum is essential for selectively exciting inter‐modal scattering and achieving strong nonreciprocity.

Finally, we also tested the SWRF modulator with optical mode sets at different wavelengths in the telecom band and confirmed the clean nonreciprocal behavior (Figure 5). We note that, due to dispersion, the frequency separation between the optical modes does vary slightly with wavelength, so the modulation frequency was accordingly adjusted from 15.5 to 17.1 GHz. The appearance of clear anti‐Stokes and Stokes sidebands in the counter‐propagating case confirms strong and highly selective inter‐modal scattering, in contrast to reduced scattering in the co‐propagating case. The enhanced phase matching provided by the SWRF structure remains effective despite the dispersion.

**FIGURE 5:**
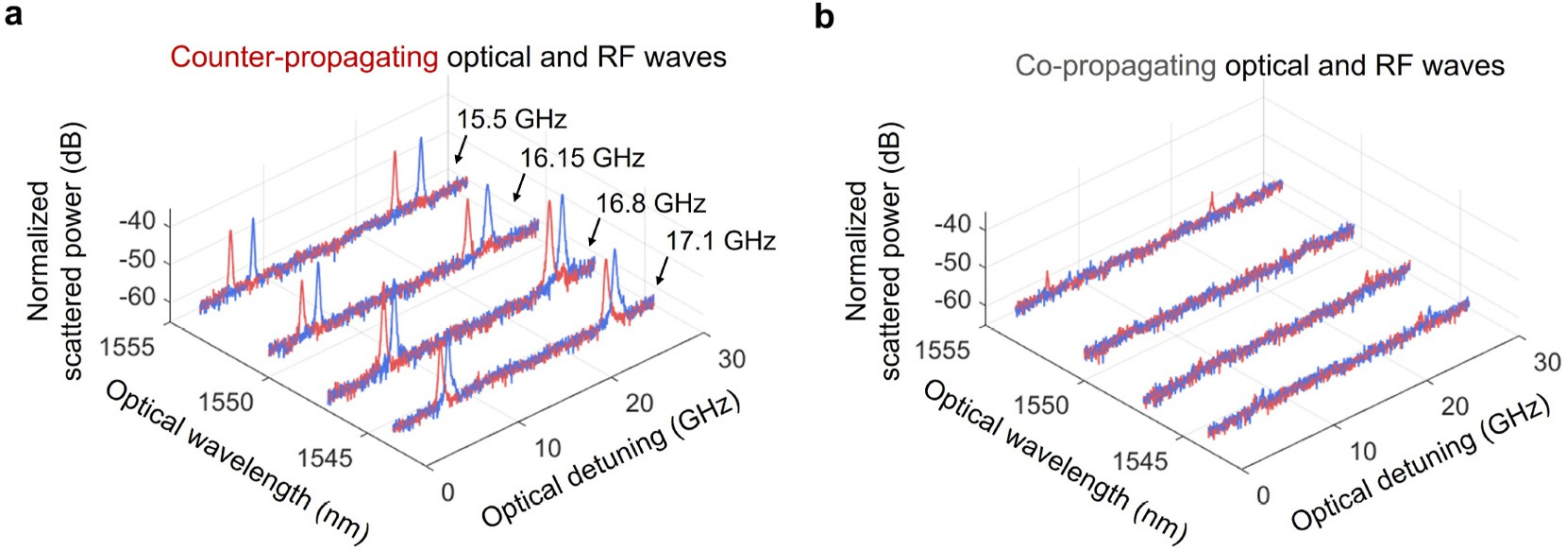
Experimental characterization of nonreciprocal scattering via the SWRF waveguide at different optical wavelengths. (a) Measured Stokes (red) and anti‐Stokes (blue) sidebands when optical and RF waves are counter‐propagating. (b) Measured Stokes (red) and anti‐Stokes (blue) sidebands when optical and RF waves are co‐propagating. Scattered power remains significantly suppressed, confirming robust nonreciprocal behavior across all tested wavelengths.

## 3 Discussion

In this work, we demonstrate a new approach to single input electro‐optic nonreciprocal intermodal scattering enabled via a slow wave radiofrequency (SWRF) waveguide design. We show that a waveguide with a large RF index can natively generate the modulation momentum required to bridge the inter‐modal gap, enabling highly selective scattering without needing multi‐stimulus configurations. In addition, we show that the finite interaction length provides a complementary degree of freedom that enables a simultaneous optimization of peak scattering for positive optical momenta and scattering nulls for negative momenta. This momentum‐engineering framework can be extended to longer modulators and higher operating frequencies, as long as the electrode interdigitation period remains deep‐subwavelength so that the transmission line behaves as an effectively uniform slow‐wave medium. Looking beyond nonreciprocal photonics, momentum‐engineered RF waves can enhance phase matching and spectral selectivity in integrated transducer designs, advancing microwave–optical transduction [36, 37, 38], a process central to quantum networking and coherent RF–photonics interfaces. This work opens new opportunities for RF‐driven isolators, circulators, nonreciprocal mode converters, and other devices leveraging momentum‐engineered RF waves in next‐generation electro‐optic systems.

## 4 Methods

### 4.1 Device Fabrication

The optical resonator and waveguides were first patterned on X‐cut thin‐film lithium niobate on a silicon dioxide layer on a silicon handle wafer using electron beam lithography. The developed pattern was then etched to a depth of 380 nm using argon‐based inductively coupled plasma reactive ion etching (ICP‐RIE). A 400‐nm thick gold electrode was subsequently fabricated on top of the 2‐μm thick silicon dioxide cladding through electron beam lithography followed by a lift‐off process. After the device is fully fabricated on the chip, the edge of the chip was cleaved to enable edge‐coupling of light from a lensed fiber to an inverse tapered waveguide.

### 4.2 Heterodyne Measurement System

We built a heterodyne interferometer to detect optical sidebands generated from the input light with GHz‐level frequency separation (see Supporting Information S1: S7). A tunable external cavity diode laser (New Focus TLB6700, 1520–1570 nm) was used as the optical source. For the reference arm, an acousto‐optic frequency shifter (Brimrose AMF‐100‐8‐1550‐2FP) was used to generate a 100‐MHz frequency‐shifted signal. The resulting optical sidebands were measured using a high‐speed photodetector (Thorlabs RXM38AF) that operates up to 38 GHz and a vector network analyzer (Rohde & Schwarz ZNA67), which also supplied the RF drive for modulation up to 18 dBm.

## Author Contributions

**Jieun Yim:** conceptualization, writing – original draft, writing – review and editing, investigation, formal analysis, data curation, methodology. **Gwan In Kim:** methodology, writing – review and editing. **Violet Workman:** methodology, writing – review and editing. **Seho Kim:** methodology, writing – review and editing. **Omar A. Barrera:** investigation, writing – review and editing, methodology. **Ruochen Lu:** methodology, writing – review and editing, funding acquisition. **Gaurav Bahl:** supervision, investigation, funding acquisition, methodology, writing – review and editing, conceptualization.

## Supporting information


Supporting Information S1

## Data Availability

The data that support the findings of this study are available from the corresponding author upon reasonable request.
