# Supplementary material for: Nonreciprocal Electro‐Optic Intermodal Scattering With Momentum Engineered RF Waves
Source: Nanophotonics. 2026 Jun 19;15(12):e70170. doi: 10.1002/nap2.70170 (PMC13642076; doi:10.1002/nap2.70170)
Supplement: Supplementary file 1 [file NAP2-15-e70170-s001.pdf]

Supplementary Information for:

Nonreciprocal Electro-Optic Intermodal Scattering with Momentum Engineered RF Waves

Jieun Yim[1*], Gwan In Kim[2], Violet Workman[3], Seho Kim[2], Omar A. Barrera[4], Ruochen Lu[4], and Gaurav Bahl[1]

[1] Department of Mechanical Science & Engineering, University of Illinois at Urbana–Champaign, Urbana, IL 61801 USA,
[2] Department of Electrical & Computer Engineering, University of Illinois at Urbana–Champaign, Urbana, IL 61801 USA,
[3] Department of Physics, University of Illinois at Urbana–Champaign, Urbana, IL 61801 USA,
[4] Department of Electrical & Computer Engineering, The University of Texas at Austin, Austin, TX 78712 USA
*jieuny@illinois.edu

S1. Scattering efficiency

Here, we present how the scattering efficiency is determined by the RF momentum spectrum. The spatial profile of the RF traveling wave that interacts with the optical field during the propagation in the y direction (for example, crystal y-axis in the main text) over the interaction length $\mathcal{L}$ (i.e. a rectangular spatial window) can be expressed as

$$f(y) = Ae^{iq_\mathrm{m}y} \cdot \mathrm{rect}(y), \quad \text{where } \mathrm{rect}(y) = \begin{cases} 1, & \text{if } 0 < y < \mathcal{L} \\ 0, & \text{otherwise} \end{cases} \tag{S1}$$

where A is the RF signal amplitude and q_m is the momentum of RF wave. By applying Fourier transform to $f(y)$, we obtain the momentum spectrum of the RF wave:

$$F(q) = A\,\mathcal{L}\,e^{-i(q-q_\mathrm{m})\mathcal{L}/2}\,\mathrm{sinc}\left[\frac{(q-q_\mathrm{m})\mathcal{L}}{2}\right]. \tag{S2}$$

This sinc function is centered at $q = q_\mathrm{m}$, which is determined by the RF index according to $q_\mathrm{m} = \frac{2\pi n_{RF}\Omega_\mathrm{m}}{c}$ (n_{RF}: effective index), and has zeros at $q_n = q_\mathrm{m} \pm \frac{2n\pi}{\mathcal{L}}$ ($n \neq 0$).

The inter-modal scattering efficiency is then defined as the square of the total electro-optic coupling rate β ($\frac{1}{s}$) between the two optical modes. For a traveling-wave modulation, the scattering efficiency scales as

$$|\beta|^2 \propto |\frac{\omega_0 \Delta n_{\mathrm{EO}}}{n_g} \cdot F(q)|^2 \tag{S3}$$

where ω_0 is the optical frequency, and n_g is the optical group index. Δn_{EO} is the electro-optically induced refractive index change, which is calculated by the overlap integral between the optical modes and the RF field via the relation [1].

$$\Delta n_{\mathrm{EO}} = \frac{-\frac{\varepsilon_0 n^5}{2}\int \left[\mathcal{E}^{\mathrm{even}}(\mathbf{r}_\perp)\right]^H \boldsymbol{r}_{\mathrm{EO}} \left[\mathcal{E}^{\mathrm{RF}}(\mathbf{r}_\perp)\right]\left[\mathcal{E}^{\mathrm{odd}}(\mathbf{r}_\perp)\right] d\mathbf{r}_\perp + \mathrm{c.c.}}{\int \left(\mathcal{E}^{\mathrm{even}}(\mathbf{r}_\perp) + \mathcal{E}^{\mathrm{odd}}(\mathbf{r}_\perp)\right)\cdot\left(\mathcal{D}^{\mathrm{even}}(\mathbf{r}_\perp) + \mathcal{D}^{\mathrm{odd}}(\mathbf{r}_\perp)\right) d\mathbf{r}_\perp} \tag{S4}$$

Here, n is the refractive index of the material, and H denotes the Hermitian conjugate. The $\boldsymbol{r}_{\mathrm{EO}}$ represents the third-rank electro-optic tensor of the material. $\mathcal{E}^{\mathrm{even}}(\mathbf{r}_\perp)$, $\mathcal{E}^{\mathrm{odd}}(\mathbf{r}_\perp)$, and $\mathcal{E}^{\mathrm{RF}}(\mathbf{r}_\perp)$ denote the transverse electric fields of the $\mathrm{TE_{even}}$, $\mathrm{TE_{odd}}$ optical modes, and the RF wave, respectively. Similarly, $\mathcal{D}^{\mathrm{even}}(\mathbf{r}_\perp)$ and $\mathcal{D}^{\mathrm{odd}}(\mathbf{r}_\perp)$ are the corresponding electric displacement fields of the optical modes. The denominator therefore represents the total electromagnetic energy normalization of the interacting modes over the transverse cross-section. Consequently, according to Eq. S3, for a fixed RF drive and RF-optical mode overlap, the electro-optically induced inter-modal scattering at any momentum spacing is governed by the squared momentum spectrum function $|F(q)|^2$.

S2. Determination of modulator length

In order to determine the ideal length $\mathcal{L}$ for the modulator, we consider the momentum spectrum $F(q)$. First, to maximize the inter-modal scattering, we should align q_{m} with Δk_{opt}, which is denoted as ① in Fig. S1a. When the relative direction of optical and RF waves is reversed, the momentum response is determined by $F(q = -\Delta k_{opt})$. To null the reverse direction response for the maximum non-reciprocity, $-\Delta k_{opt}$ should be located at a zero of the sinc function (② in Fig. S1a). Finally, to minimize intra-modal scattering, $F(q = -\Delta k_{\mathrm{opt}_{\mathrm{intramodal}}})$ should also be located near a zero (③ in Fig. S1). By substituting conditions ① and ② into Eq. (S2), we can derive the interaction length as

$$\mathcal{L} = \frac{p\pi}{q_{\mathrm{m}}} \tag{S5}$$

where p is a positive integer that can be determined arbitrarily as in Fig. S1b. In our design, we use $q_{\mathrm{m}} = 3300$ rad/m and $p = 3$ which leads to $\mathcal{L} = 2.85$ mm, by which we can also fulfill the requirement ③. Since we are interested in the non-reciprocal contrast of the spectral scattering efficiency, which is expressed as the ratio $|\beta(q=\Delta k_{\mathrm{opt}})|^2/|\beta(q=-\Delta k_{\mathrm{opt}})|^2$, we can instead compute the directly proportional ratio $|F(q=\Delta k_{\mathrm{opt}})|^2/|F(q=-\Delta k_{\mathrm{opt}})|^2$ with $\mathcal{L} = 2.85$ mm. (see Fig. S2). Since

our experiments invoke a modulation frequency range of $\Omega_{\rm m} = 16 - 20$ GHz, the corresponding modulation momentum range is $q_m = 3100 - 4100$ rad/m as described in the main text. Fig. S2 shows that, for our chosen interaction length, the non-reciprocal contrast does vary with the modulation momentum $q_{\rm m}$, but remains robustly above 20 dB across the entire range.

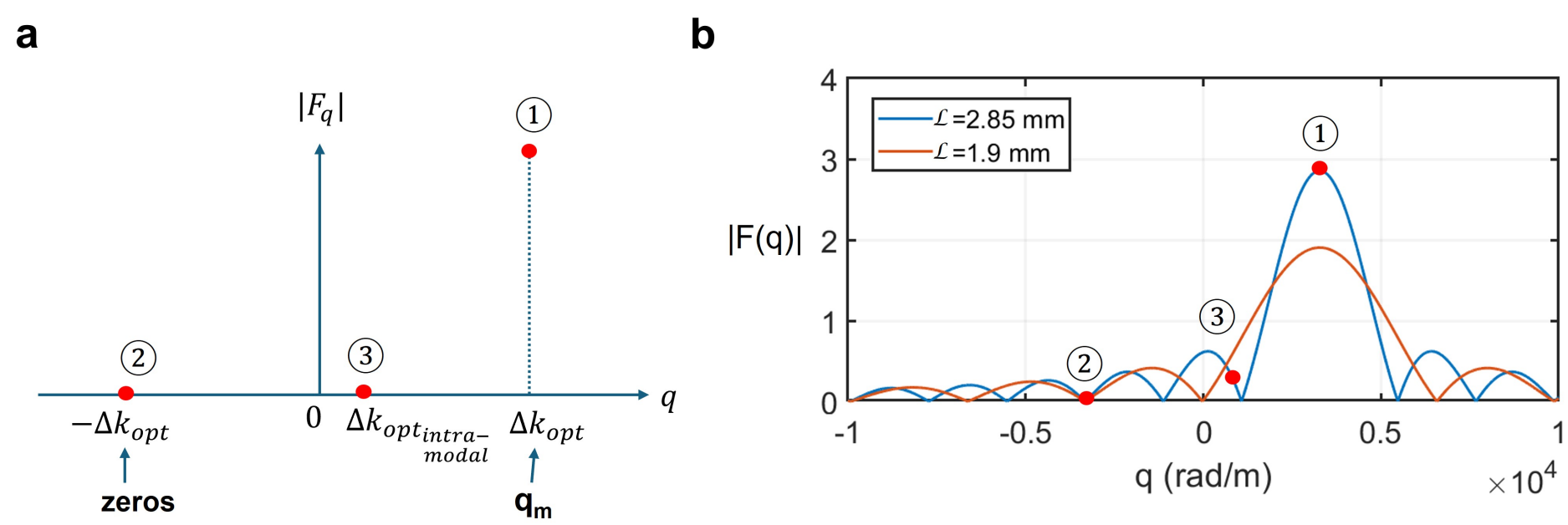

Figure S1: **(a)** Desired values that guide the momentum spectrum design. **(b)** Example momentum spectra for two different modulator lengths $\mathcal{L}$.

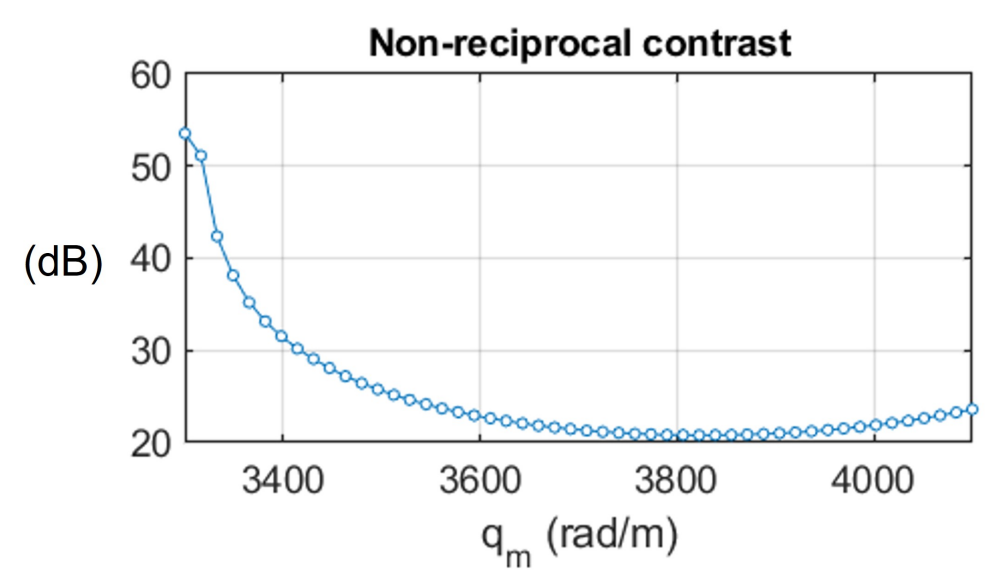

Figure S2: Non-reciprocal contrast calculated from $|F(q{=}\Delta k_{\rm opt})|^2/|F(q{=}-\Delta k_{\rm opt})|^2$ for modulator length $\mathcal{L} = 2.85$ mm within a range of modulation momentum $q_m = 3300 - 4100$ rad/m.

S3. Fundamental limit of non-reciprocal contrast due to RF attenuation

Here, we examine how the RF attenuation affects the momentum spectrum of the scattering efficiency and fundamentally limits the non-reciprocal contrast in the system. By including the attenuation constant of the RF traveling wave, α, Eq. S1 can be rewritten as:

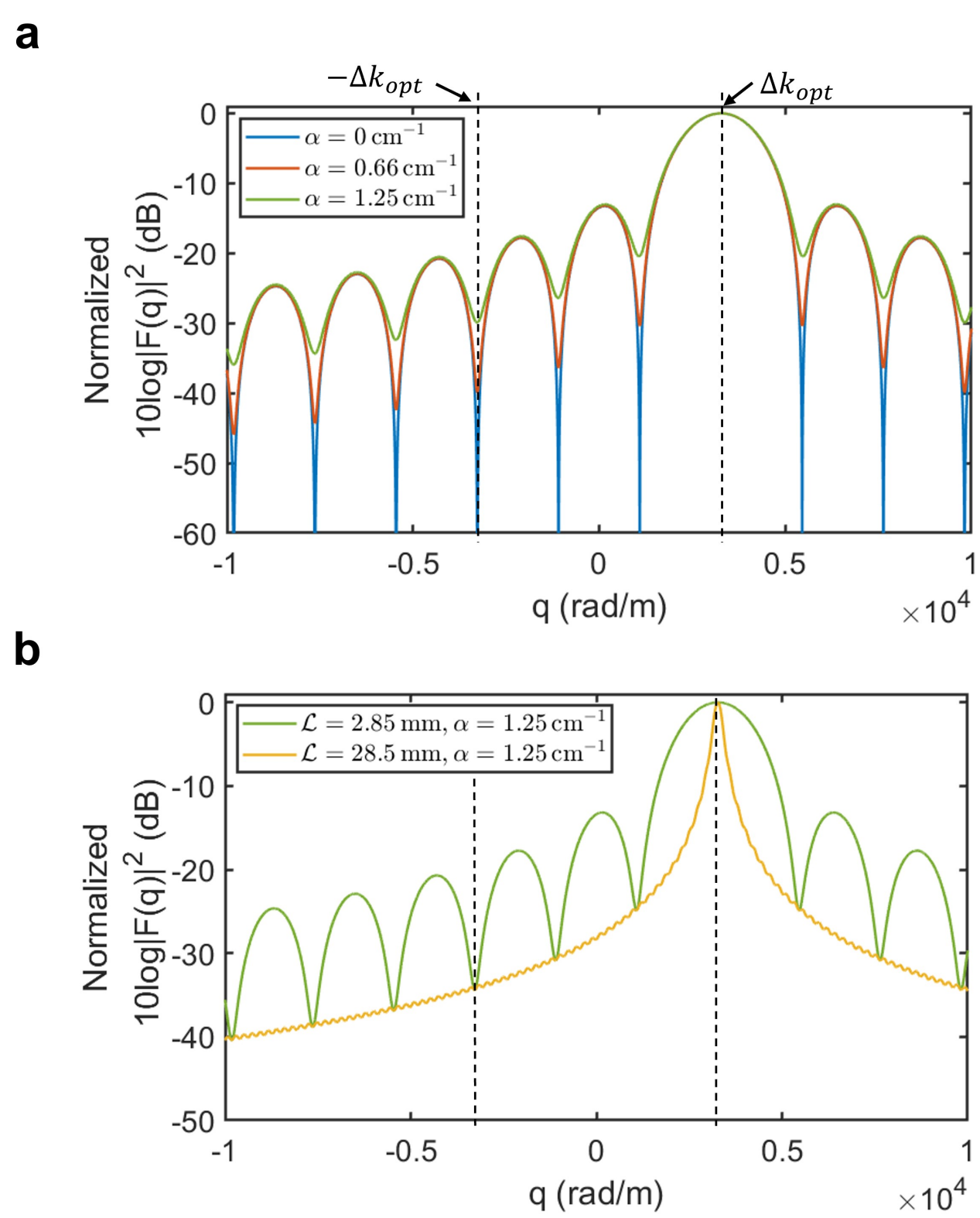

Figure S3: **(a)** Normalized spectral scattering efficiency (dB scale) for the SWRF transmission lines with $q_{\mathrm{m,SWRF}} = 3300$ rad/m and different values of attenuation constant α. **(b)** Normalized spectral scattering efficiency (dB scale) for the SWRF transmission lines with $q_{\mathrm{m,SWRF}} = 3300$ rad/m of different interaction lengths $\mathcal{L}$ and the same attenuation constant α.

$$f(y) = Ae^{(-\alpha+iq_{\mathrm{m}})y} \cdot \mathrm{rect}(y), \quad \text{where } \mathrm{rect}(y) = \begin{cases} 1, & \text{if } 0 < y < \mathcal{L} \\ 0, & \text{otherwise} \end{cases} \tag{S6}$$

By applying Fourier transform to $f(y)$, we obtain the momentum spectrum of the RF wave:

$$F(q) = A\frac{1 - e^{-(\alpha+i(q-q_{\mathrm{m}}))\mathcal{L}}}{\alpha + i(q - q_{\mathrm{m}})} \tag{S7}$$

Under the perfect momentum matching condition $q_{\mathrm{m}} = \Delta k_{\mathrm{opt}}$, the non-

reciprocal contrast is

$$\left|\frac{F(q=\Delta k_{\text{opt}})}{F(q=-\Delta k_{\text{opt}})}\right|^2 = \frac{\left(1-e^{-\alpha\mathcal{L}}\right)^2}{\alpha^2}\cdot\frac{\alpha^2+4q_{\text{m}}^2}{1+e^{-2\alpha\mathcal{L}}-2e^{-\alpha\mathcal{L}}\cos\left(2q_{\text{m}}\mathcal{L}\right)} \tag{S8}$$

In the lossless case (Supplementary §S2), we had calculated the preferred interaction length as $\mathcal{L} = p\pi/q_{\text{m}}$ (Eq. S5) to enforce a spectral null at $q = -\Delta k_{\text{opt}}$. Under this condition we find that Eq. S8 is simplified to:

$$\left|\frac{F(q=\Delta k_{\text{opt}})}{F(q=-\Delta k_{\text{opt}})}\right|^2 = 1+\frac{4q_{\text{m}}^2}{\alpha^2} \tag{S9}$$

Here we see that in the case where the interaction length is chosen to satisfy the spectral-null condition, the non-reciprocal contrast becomes solely determined by the ratio q_{m}/α. To visually show the effect of RF attenuation, we plot the normalized $|F(q)|^2$ for the SWRF transmission line in Fig. S3a with different values of α. When $\alpha = 0$, a spectral null is formed at $q = -\Delta k_{\text{opt}}$ while maintaining a maximum at $q = +\Delta k_{\text{opt}}$, enabling arbitrarily large non-reciprocal contrast. In the presence of RF attenuation, the traveling-wave amplitude decays along the propagation direction, preventing the formation of an exact spectral null, allowing some residual scattering in the nominally suppressed direction. The role of interaction length is shown in Fig. S3b. Although increasing $\mathcal{L}$ (while satisfying $\mathcal{L} = p\pi/q_{\text{m}}$) sharpens the spectral response, the suppression of scattering at $q = -\Delta k_{\text{opt}}$ is limited at the same level as predicted from Eq. S9. According to Eq. S9, we can estimate the theoretical upper bound on the non-reciprocal contrast achievable with our SWRF device. Using the general line–line method [2] (details in Supplementary §S6), the attenuation constant of the SWRF transmission line was calculated to be 1.25 cm^{-1} (10.86 dB/cm). This attenuation limits the maximum achievable non-reciprocal contrast to 34.5 dB even under perfect momentum-matching conditions. Eq. S9 also allows us to estimate the RF attenuation required for a targeted contrast. For example, for $q_{\text{m}} = 3300$ rad/m, achieving a non-reciprocal contrast of 40 dB requires $\alpha < 0.66\ \text{cm}^{-1}$ (5.7 dB/cm). Since increasingly complex electrode structures generally introduce larger RF losses, achieving higher contrast requires reducing RF attenuation through improved electrode designs and lower-loss materials.

S4. Dual-waveguide design

Mode analysis was performed in COMSOL Multiphysics to design the dual-waveguide geometry. The optimized cross section of the dual optical waveguide is shown in Fig. S4b. Several practical constraints were incorporated in the design. The optical waveguide separation must be sufficiently small to enable evanescent coupling that produces a resolvable splitting between the TE_{even} and TE_{odd} modes in the transmission spectrum, even in the presence of finite linewidths set by the loaded quality factors. At the same time, the optical waveguide width cannot be excessively large, as this would support higher-order

modes. Conversely, the optical waveguide width cannot be too small, and the lithium niobate etch depth cannot be excessively deep, since increased optical mode interaction with etched sidewalls degrades the optical quality factor. Consequently, both design and fabrication constraints were jointly considered in selecting the final geometry.

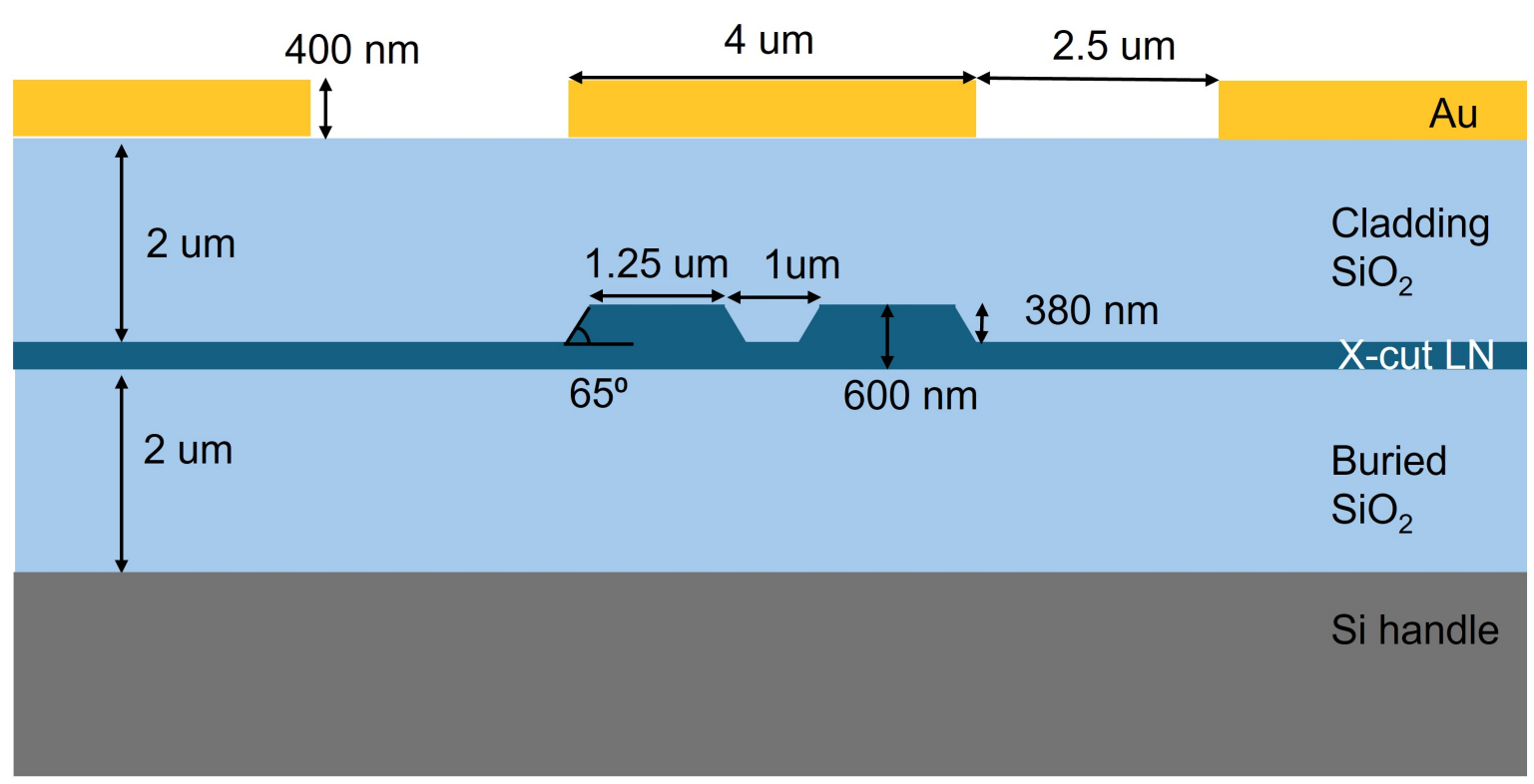

Figure S4: Cross-sectional schematic of the double waveguide system and electrodes used in our experiments.

S5. Dimensions of electrodes

Through the design principle described in the main text, we performed 3D simulations and optimized the design of the electrode. The electrode thickness was set to 400 nm. The dimensions of the SWRF and CPW electrodes used in our device are shown in Fig. S5.

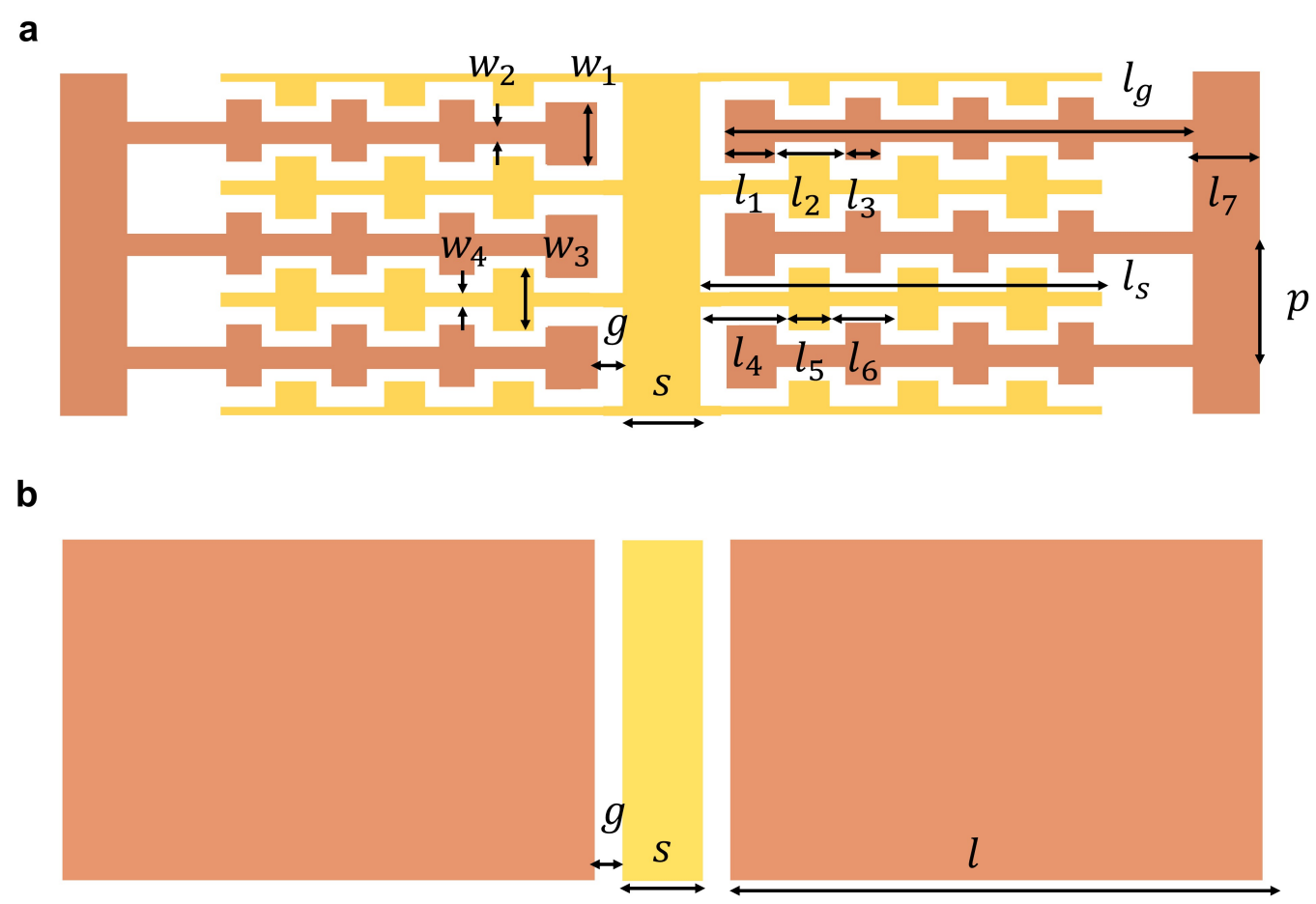

Figure S5: Dimensions of **(a)** SWRF **(b)** CPW electrodes. The specific dimensions are listed: $s = 4$ µm, $g = 2.5$ µm, $p = 10$ µm, $l = 180$ µm, $l_g = 170$ µm, $l_1 = 4.5$ µm, $l_2 = 7$ µm, $l_3 = 3$ µm, $l_4 = 8$ µm, $l_5 = 5$ µm, $l_6 = 5$ µm, $l_7 = 10$ µm, $w_1 = 7$ µm, $w_2 = 3$ µm, $w_3 = 5$ µm, $w_4 = 1$ µm.

S6. General line-line method

To extract the effective RF index of the transmission lines, we employ the general line–line method [2]. Here, two transmission lines with identical cross-sectional geometry (SWRF or CPW) are fabricated, differing only in physical length. Under this condition, the propagation constant can be extracted directly from the length difference. Let the two lines have lengths $\mathcal{L}_1$ and $\mathcal{L}_2$, with $\Delta\mathcal{L} = \mathcal{L}_2 - \mathcal{L}_1$. Their measured wave–cascade matrices are denoted M_1 and M_2, referenced to the same reference impedance (50 Ω). These matrices can be expressed as

$$M_1 = X\,T(\mathcal{L}_1)\,Y, \tag{S10}$$

$$M_2 = X\,T(\mathcal{L}_2)\,Y, \tag{S11}$$

where X and Y represent input and output networks (including RF probes, cables, and pads), and $T(\mathcal{L})$ is the wave–cascade matrix of the uniform transmission line section.

Forming the product as

$$M = M_2 M_1^{-1} = X\,T(\mathcal{L}_2)T(\mathcal{L}_1)^{-1}X^{-1}, \tag{S12}$$

eliminates the unknown network Y. Due to the trace invariance,

$$\mathrm{Tr}(M_2 M_1^{-1}) = \mathrm{Tr}(T(\mathcal{L}_2)T(\mathcal{L}_1)^{-1}). \tag{S13}$$

For a uniform transmission line of length $\mathcal{L}$ and complex propagation constant γ, the corresponding wave–cascade matrix has eigenvalues $e^{\pm\gamma\mathcal{L}}$, associated with the forward- and backward-propagating waves. Consequently, for the length difference $\Delta\mathcal{L}$,

$$\mathrm{Tr}(M) = e^{\gamma\Delta\mathcal{L}} + e^{-\gamma\Delta\mathcal{L}} = 2\cosh(\gamma\Delta\mathcal{L})\,. \tag{S14}$$

The complex propagation constant is thus extracted as

$$\gamma(\omega) = \frac{1}{\Delta\mathcal{L}}\cosh^{-1}\left(\frac{\mathrm{Tr}(M)}{2}\right), \tag{S15}$$

where the branch of the inverse hyperbolic cosine is chosen such that $\mathrm{Re}[\gamma] \geq 0$. The RF effective index is then obtained from the imaginary part of γ,

$$n_{\mathrm{RF}}(\omega) = \frac{\mathrm{Im}[\gamma(\omega)]}{\omega/c}. \tag{S16}$$

S7. Heterodyne measurement and normalization

As described in Methods in the main text, we build a heterodyne setup (Fig. S6) to measure the scattered sideband powers relative to the input light, capable of measurements up to 26 GHz.

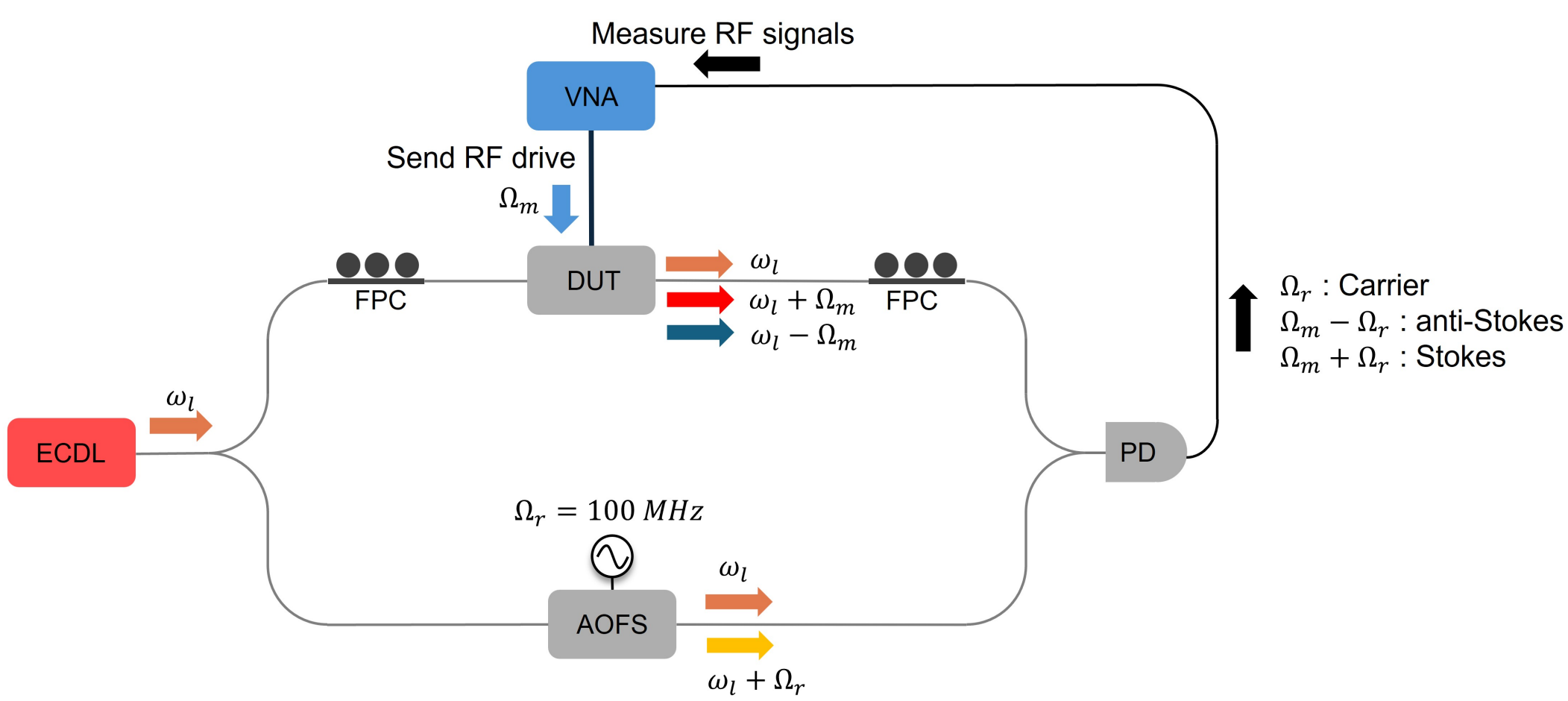

Figure S6: Schematic of the heterodyne detection setup used to characterize anti-Stokes and Stokes sidebands. ECDL: External cavity diode laser, DUT: device under test, VNA: Vector network analyzer, FPC: Fiber polarization controller, AOFS: Acousto-optic frequency shifter. PD: Photodetector. ω_l: Laser input frequency, Ω_r: reference frequency, Ω_m: RF modulation frequency. The direction of RF modulation can be reversed by interchanging the transmitting and receiving ports of the VNA. All the RF signals detected by PD were measured by the VNA.

At the photodetector (PD), the detected optical field contains contributions from the laser output together with the anti-Stokes (AS), Stokes (S), and reference tones. Therefore, the optical field that arrives at the PD is described as

$$E(t) = S_{\mathrm{out},0}e^{-i\omega_l t} + S_{\mathrm{out},+1}e^{-i(\omega_l+\Omega_\mathrm{m})t} + S_{\mathrm{out},-1}e^{-i(\omega_l-\Omega_\mathrm{m})t} + S_\mathrm{r}e^{-i(\omega_l+\Omega_\mathrm{r})t} \tag{S17}$$

Here, $S_{\mathrm{out},0}$, $S_{\mathrm{out},+1}$, and $S_{\mathrm{out},-1}$ denote the amplitudes of the carrier, AS, and S optical fields, and their oscillating frequencies are ω_l, $\omega_l + \Omega_\mathrm{m}$, and $\omega_l - \Omega_\mathrm{m}$, respectively. S_r denotes the amplitude of the reference field oscillating at $\omega_l + \Omega_r$. These different frequency components and their pathways are illustrated in Fig. S6. Since the photocurrent generated by the photodetector is proportional to the incident optical power, $i(t) \propto |E(t)|^2$, interference between the reference field and the carrier transmission and first-order AS and S sidebands produces distinct RF beat-note photocurrent components at the corresponding beat frequencies as

$$i_{\Omega_\mathrm{r}}(t) \propto 2\Re\{S_{\mathrm{out},0}S_\mathrm{r}^* e^{i(\Omega_\mathrm{r})t}\} \tag{S18}$$

$$i_{\Omega_\mathrm{m}-\Omega_\mathrm{r}}(t) \propto 2\Re\{S_{\mathrm{out},+1}S_\mathrm{r}^* e^{-i(\Omega_\mathrm{m}-\Omega_\mathrm{r})t}\} \tag{S19}$$

$$i_{\Omega_\mathrm{m}+\Omega_\mathrm{r}}(t) \propto 2\Re\{S_{\mathrm{out},-1}S_\mathrm{r}^* e^{-i(\Omega_\mathrm{m}+\Omega_\mathrm{r})t}\} \tag{S20}$$

As a result, the carrier transmission, AS and S signal powers can be acquired at the vector network analyzer (assuming total scaling factor including PD gain to be g_pd) as

$$P_{\Omega_\mathrm{r}} = g_\mathrm{pd}|S_{\mathrm{out},0}|^2|S_\mathrm{r}|^2 \tag{S21}$$

$$P_{\Omega_\mathrm{m}-\Omega_\mathrm{r}} = g_\mathrm{pd}|S_{\mathrm{out},+1}|^2|S_\mathrm{r}|^2 \tag{S22}$$

$$P_{\Omega_\mathrm{m}+\Omega_\mathrm{r}} = g_\mathrm{pd}|S_{\mathrm{out},-1}|^2|S_\mathrm{r}|^2 \tag{S23}$$

To normalize the measured signals with respect to the optical input into the device, we measure the carrier transmission at the vector network analyzer to obtain

$$P_\mathrm{in} = g_\mathrm{pd}|S_\mathrm{in}|^2|S_\mathrm{r}|^2, \tag{S24}$$

where S_in denotes the optical field amplitude inside the bus waveguide. This quantity can be obtained directly from the transmission spectrum (assuming low optical loss) when the carrier laser is tuned away from any optical modes, since at that point the carrier transmission satisfies $S_{\mathrm{out},0} = S_\mathrm{in}$. By normalizing Eqs. S21–S23 with P_in, we obtain the expression for the normalized carrier signal and AS and S sideband powers as:

$$P_{\Omega_\mathrm{r},\mathrm{norm}} = \frac{|S_{\mathrm{out},0}|^2}{|S_\mathrm{in}|^2} \tag{S25}$$

$$P_{\Omega_\mathrm{m}-\Omega_\mathrm{r},\mathrm{norm}} = \frac{|S_{\mathrm{out},+1}|^2}{|S_\mathrm{in}|^2} \tag{S26}$$

$$P_{\Omega_\mathrm{m}+\Omega_\mathrm{r},\mathrm{norm}} = \frac{|S_{\mathrm{out},-1}|^2}{|S_\mathrm{in}|^2} \tag{S27}$$

To see how the normalized sideband power represents the scattering efficiency, we can derive the full expression for Eqs. (S26) and (S27), according to coupled-mode theory [3] as:

$$P_{\Omega_\mathrm{m}-\Omega_\mathrm{r},\mathrm{norm}} = \left| \frac{i\beta\sqrt{\kappa_{\mathrm{ex1}}\kappa_{\mathrm{ex2}}}}{\left(\frac{\kappa_2}{2} - i(\omega_l - \omega_2 + \Omega_\mathrm{m})\right)\left(\frac{\kappa_1}{2} - i(\omega_l - \omega_1)\right)} \right|^2 \tag{S28}$$

$$P_{\Omega_\mathrm{m}+\Omega_\mathrm{r},\mathrm{norm}} = \left| \frac{i\beta^*\sqrt{\kappa_{\mathrm{ex1}}\kappa_{\mathrm{ex2}}}}{\left(\frac{\kappa_1}{2} - i(\omega_l - \omega_1 - \Omega_\mathrm{m})\right)\left(\frac{\kappa_2}{2} - i(\omega_l - \omega_2)\right)} \right|^2 \tag{S29}$$

Here $\kappa_{1,2}$, $\kappa_{\mathrm{ex}1,2}$ and $\omega_{1,2}$ are total loss, extrinsic loss and resonant frequencies of the coupled optical modes (i.e. $\mathrm{TE_{even}}$ and $\mathrm{TE_{odd}}$ in the resonator). Therefore, the normalized scattered powers of the anti-Stokes and Stokes sidebands are proportional to scattering efficiency $|\beta|^2$.

S8. Robustness and spectral selectivity of non-reciprocal scattering

As discussed in the main text, the RF index and interaction length of the SWRF electrode were designed to provide an effective RF momentum in the range of $q_{\mathrm{m,SWRF}} = 3300$–$4100$ rad/m over $\Omega_\mathrm{m} = 16$–20 GHz. Experimentally, however, the extracted value is $q_{\mathrm{m,SWRF}} = 3240$ rad/m at $\Omega_\mathrm{m} = 16.8$ GHz, which is slightly outside the targeted range. Here we show that robust non-reciprocal scattering is still obtained despite this discrepancy.

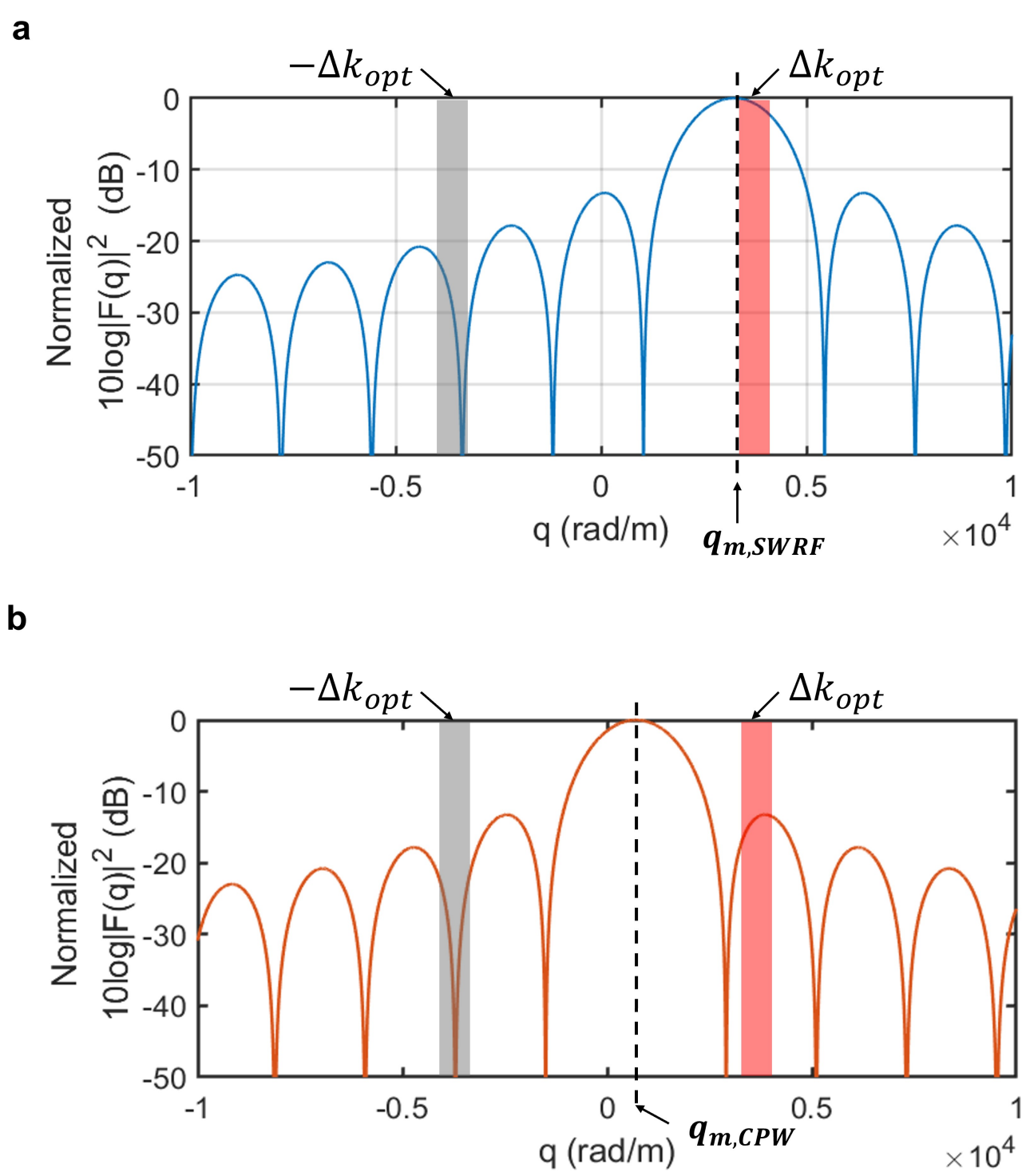

Figure S7: Normalized spectral scattering efficiency (dB scale) for the **(a)** SWRF and **(b)** CPW transmission lines for RF drive frequency $\Omega_\mathrm{m} = 16.8$ GHz and interaction length $\mathcal{L} = 2.85$ mm. In each case, the efficiency peaks at the modulation momenta $q_\mathrm{m,SWRF}$ and $q_\mathrm{m,CPW}$. The ranges of Δk_opt and $-\Delta k_\mathrm{opt}$, corresponding to the optical momentum gaps for counter-propagating and co-propagating cases, are highlighted by the red and gray shaded regions.

Fig. S7a shows the normalized finite-interaction-length spectral scattering efficiency for $q_\mathrm{m,SWRF} = 3240$ rad/m, plotted on a dB scale. As established in Supplementary §S7, the normalized scattered power P detected in the heterodyne measurement is proportional to $|F(q)|^2$. Consequently, a 20 dB non-reciprocity observed in the measured scattered power between the counter-propagating and co-propagating configurations corresponds directly to a 20 dB asymmetry in the spectral scattering efficiency, i.e., between $|F(q = \Delta k_\mathrm{opt})|^2$ and $|F(q = -\Delta k_\mathrm{opt})|^2$. From the designed optical dispersion, for a frequency separation of $\Delta\omega_\mathrm{opt} = 16.8$ GHz, the optical momentum gap Δk_opt spans 3340–4150 rad/m over the laser tuning range of 1520–1570 nm. Within this range, the SWRF transmission line exhibits robust non-reciprocal contrast between the counter-propagating and co-propagating cases, with a worst case value of $18 - 19$ dB.

For comparison, Fig. S7b shows the normalized finite-interaction-length spec-

tral scattering efficiency of the CPW transmission line. Since $q_{\mathrm{m,CPW}} = 704\,\mathrm{rad/m}$, the ranges of Δk_{opt} and $-\Delta k_{\mathrm{opt}}$ fall within higher-order lobes of the sinc-shaped spectral response. As a result, although the non-reciprocal contrast depends on the specific choice of finite interaction length, the non-reciprocity achievable with the CPW transmission line over a broad optical wavelength range is intrinsically more limited. In this case, the worst case non-reciprocal contrast between the counter-propagating and co-propagating cases over the laser tuning range of 1520–1570 nm is around 5 dB, which is substantially smaller than what may be achieved with the SWRF transmission line.

Supplementary References

[1] Örsel, O. E. & Bahl, G. Electro-optic non-reciprocal polarization rotation in lithium niobate. *APL Photonics* **8**, 096107 (2023).

[2] Bao, X. *et al.* A General Line–Line Method for Dielectric Material Characterization Using Conductors With the Same Cross-Sectional Geometry. *IEEE Microwave and Wireless Components Letters* **28**, 356–358 (2018).

[3] Sohn, D. B., Kim, S. & Bahl, G. Time-reversal symmetry breaking with acoustic pumping of nanophotonic circuits. *Nature Photonics* **12**, 91–97 (2018).
